# Adsorption Performance for Chromium(VI) of a UiO-66-Ce Metal–Organic Framework Built by DL-Aspartic Acid

**DOI:** 10.3390/ma17215293

**Published:** 2024-10-30

**Authors:** Xiaoyi Lin, Sabrina Yanan Jiang, Gang Li

**Affiliations:** 1 National Observation and Research Station of Coastal Ecological Environments in Macao, Macao Environmental Research Institute, Macau University of Science and Technology, Macao 999078, China; 2230028761@must.edu.mo; 2 College of Chemistry, Zhengzhou University, Zhengzhou 450001, China

**Keywords:** metal–organic framework, cerium(IV), chromium(IV), adsorption

## Abstract

Metal–organic frameworks (MOFs) have recently received a lot of interest for their use in adsorbing and eliminating hexavalent chromium from water. Obtaining low-cost, biocompatible, and environmentally friendly MOFs for research in this field is vital. One very stable three-dimensional UiO-66-Ce(IV) MOF, **Ce-asp**, was synthesized with a high yield using an amino acid ligand, DL-aspartic acid. As a result, the adsorption characteristics of the MOF against hexavalent chromium ions in aqueous solution were examined. The effects of time, solution pH, MOF dose, and beginning chromium(VI) content in aqueous solution were investigated on adsorption. More crucially, the adsorption mechanism of this MOF for chromium(VI) was proposed, setting the groundwork for its future use in chromium(VI) removal in real-world waters.

## 1. Introduction

Chromium is a usual heavy metal in wastewater, primarily in the form of Cr(VI) and Cr(III) in the environment. Hexavalent chromium exhibits acute toxicity, slow degradation, significant migration, easy accumulation, and carcinogenicity, with a toxicity 500 times that of trivalent chromium [1]. Heavy metals, unlike organic molecules, cannot be biodegraded and accumulate in the human body at a certain quantity, posing a threat to human health. Furthermore, chromium-containing wastewater poses a substantial threat to aquatic biological ecosystems [2,3,4]. As a result, one of the most pressing issues is determining ways to reduce hexavalent chromium concentrations in wastewater.

Currently, the most prevalent methods for removing Cr(VI) from wastewater include electrochemical methods, adsorption, ion exchange, photocatalysis, and membrane separation [5,6,7,8]. Adsorption is one of the most promising methods owing to its high efficiency, low cost, and simplicity of operation [9,10,11,12]. The adsorbent is the key to removing heavy metals from water via the adsorption method. The properties of different heavy metals in wastewater might be highly diverse, so it is very crucial to pick acceptable adsorption materials.

In this context, metal–organic frameworks (MOFs) have attracted much attention regarding the removal of different metal ions [13,14,15,16,17,18,19], including the adsorption of Cr(IV) ions in water [20,21,22,23,24]. MOFs are crystalline porous materials generated by the coordination bonds that connect metal nodes with organic ligands [25,26] and exhibit exceptional features, such as a big specific surface area, tunable pore sizes, vast porosity, high topological differences, and excellent hydrothermal stability. This has provided a solid material foundation for their application as a hexavalent chromium ion adsorbent [27,28,29]. With the rapid advancement of crystal engineering technology in recent years, a considerable number of MOFs with high water and chemical stability have been created, and they have demonstrated great application potential in a variety of domains, particularly as adsorbents. For example, recently, according to the hard–soft acid–base (HSAB) theory, one important type of MOF assembled by O ligands/carboxylate ligands and high-priced metal cations (Zr^4+^, Hf^4+^, and Ce^4+^) has been formed with high stability, such as the UiO-66-type MOF formed by zirconium(IV) ions and terephthalic acid, which demonstrates super stability [30].

Although various zirconium(IV)-based MOFs have been employed for the adsorption of hexavalent chromium ions [31,32,33,34], research on cerium(IV)-based MOFs is limited. Furthermore, the use of MOFs derived from amino acid ligands has recently attracted a lot of attention. Such MOFs demonstrate a wide range of application owing to their high biocompatibility and low cost [35,36,37].

Motivated by this, in this paper, we refer to the method of study [38], using cerium(IV) ion and an amino acid ligand, DL-aspartic acid in aqueous solution, employing formic acid as the regulator, to obtain a MOF equal to UiO-66 at a high yield and carry out an adsorption study of hexavalent chromium ion. In addition, compared to UiO-66-series MOFs built with terylene acid and its derivatives, UiO-66-Ce MOF offers the benefits of low cost, good biocompatibility, easy synthesis, and mass production due to the utilization of a cheap natural product, DL-aspartic acid ligand. It will provide an adequate material basis for future practical applications.

A systematic study shows that the MOF **Ce-asp** performs better Cr(IV) adsorption in aqueous solution. Langmuir isothermal adsorption and pseudo-second-order adsorption models could describe the chromium(VI) adsorption behavior by the MOF, laying a foundation for subsequent application research.

## 2. Materials and Methods

### 2.1. Materials

The reagents were all purchased from McLean Reagent Company (Shanghai, China) or China Scientific Research Reagent Company (Shanghai, China) and are pure analytical goods. Unless otherwise specified, no further purification was carried out. Ammonium ceric nitrate ((NH_4_)_2_Ce(NO_3_)_6_, 99.0%), DL-aspartic acid (HOOCCH_2_CH(NH_2_)COOH, 98.0%), formic acid (HCOOH, 99.0%), potassium dichromate (K_2_Cr_2_O_7_, 99.8%), hydrochloric acid (HCl, 38%), and sodium hydroxide (NaOH, 98.0%) were used. Deionized H_2_O was adopted. The ultraviolet–visible spectrum was determined by a TU-1901 ultraviolet–visible spectrophotometer (Beijing, China).

### 2.2. Determinations

A Nicolet NEXUS 470-FTIR analyzer was used to obtain the infrared spectrum (KBr, 400–4000 cm^−1^) (Kyoto, Japan). A Rigaku D/MAX-3 diffractometer (Cu target; λ = 1.5418 Å) was used to obtain PXRD patterns (Rigaku Corporation, Akishima, Japan). Using a NETZSCH STA 409PC analyzer (air flow rate: 10 °C/min) (NETZSCH Corporation, Selb, Germany), TGA was performed. The ASAP 2420 apparatus provided the isothermal adsorption/desorption of N_2_ (−196 °C) (Micromeritics Corporation, Norcross, GA, USA).

### 2.3. Preparation of MOF **Ce-Asp**

We primarily relied on the approach reported in reference [38] to synthesize **Ce-asp**: 319 mg (2.4 mmol) of DL-aspartic acid was firstly dissolved in 9 mL of H_2_O, and then 9 mL of formic acid and 3 mL of ammonium cerium nitrate aqueous solution (0.533 mol/L) were added. The mixture was heated and stirred at 100 °C for 0.5 h and cooled to 25 °C. The crystalline products of **Ce-asp** were collected using centrifugation (10,000 rpm/min), washed twice with acetone, and then dried under vacuum at 70 °C for 12 h. Anal. Calcd for Ce_6_C_24_O_59_H_88_N_6_: C: 12.84%; H: 3.95%; N: 3.74%. Found: C: 12.68%; H: 3.74%; N: 3.53%. It yielded 87%.

### 2.4. Stability Measurements

First, an adequate amount of the MOF sample was soaked in deionized water for seven days at room temperature or refluxed in deionized water for 24 h, filtered and dried in the air, and then a PXRD test was performed to ensure its water stability. Second, MOF samples were immersed in HCl or NaOH solutions of varying pH values for 24 h, filtered, washed with H_2_O, air-dried, and then PXRD evaluated to ensure chemical stability.

### 2.5. Cr(IV) Adsorption Determinations

The stock solution of 100 mg/L Cr(IV) was prepared by dissolving K_2_Cr_2_O_7_ (0.283 g) in H_2_O (1000.0 mL). Subsequently, the solution with the required concentration of chromium(IV) was obtained by diluting the stock solution. The NaOH (0.1 mol/L) or HCl (0.1 mol/L) aqueous solutions were employed to modify the pH values of the test solutions.

The main procedure of the adsorption experiment was as follows: To begin, 0.40, 0.60, 1.00, 2.00, 3.00, 4.00, 6.00, 8.00, and 10.0 mL of Cr(IV) standard solutions (100 mg/L) were introduced to a series of 10 mL colorimetric tubes and diluted to the line with water. The solution’s absorbance was then measured at 350 nm using a UV–VIS spectrophotometer (Beijing, China), and a standard working curve of absorbance vs. hexavalent chromium content was constructed (Figure S1). Then, the MOF was accurately weighed (*m*) and placed in a triangle bottle with a specific volume (*V*) of a certain concentration (*C*_0_) of Cr(IV) solution. This was stirred for a certain time (*t*) after sampling, and then centrifuged to separate the solid. The clarified liquid was then measured with a UV–VIS spectrophotometer at 350 nm to determine the absorbance. In addition, the concentration (*C*_1_) of the adsorbed solution was derived according to the measured absorbance and the above standard working curve. Equations (1) and (2) were used to compute the adsorption capacity (*Q*_e_, mg/g) and removal rate (*R*, %) of the MOF for Cr(IV).

*Q*_e_ = [(*C*_0_ − *C*_1_) × *V*]/*m* (1)

*R =* (*C*_0_ − *C*_1_)/*C_0_* × 100% (2)

Here, *V* is the volume of Cr(IV) solution (mL); *m* is the mass (mg) of the adsorbent (MOF); and *C*_0_ and *C*_1_ are the mass concentration (mg/L) of Cr(IV) solution at the initial and time *t*, respectively.

### 2.6. Adsorption Kinetics Exploration

The adsorption kinetics test was performed by injecting 10 mg of **Ce-asp** into 10 mL Cr(VI) solution (100 mg/L), and the adsorption time was set from 0 to 48 h. The quasi-first-order and quasi-second-order kinetic models were obtained by linear fitting to study the adsorption mechanism of the MOF on Cr(VI). The linear equations are shown in (3) and (4), respectively:

(3) $$\mathrm{Ln}(Q_{e}-Q_{t})=\mathrm{Ln}Q_{e}-k_{1}t$$

(4) $$\frac{t}{Q_{t}}=\frac{1}{k_{2\;}Q_{e}^{2}}+\frac{t}{Q_{e}}$$

Here, *Q_t_* is the adsorption capacity at time *t* (mg/g); *k*_1_ is the rate constant (h^−1^) of the quasi-first-order model; and *k*_2_ is the rate constant of the quasi-second-order model (h·g/mg).

### 2.7. Adsorption Isotherm Exploration

The adsorption isotherm was simulated by the Langmuir model (5) and the Freundlich model (6).

(5) $$Q_{e\;}=\frac{Q_{m}k_{3}C_{e}}{1+k_{3}C_{e}}$$

(6) $$Q_{e}=k_{4}C_{e}^{n}$$

Here, *Q_m_* is the maximum adsorption capacity (mg/g); *C_e_* is the concentration of the solution at adsorption equilibrium (mg/g); *k*_3_ is the Langmuir model constant (L/mg); *k*_4_ is the adsorption capacity of the Freundlich model (mg^(1−n)^·L^n^·g^−1^); and n is the Freundlich model constant.

## 3. Results

### 3.1. Characterizations and Structural Features of **Ce-Asp**

**Figure 1:**
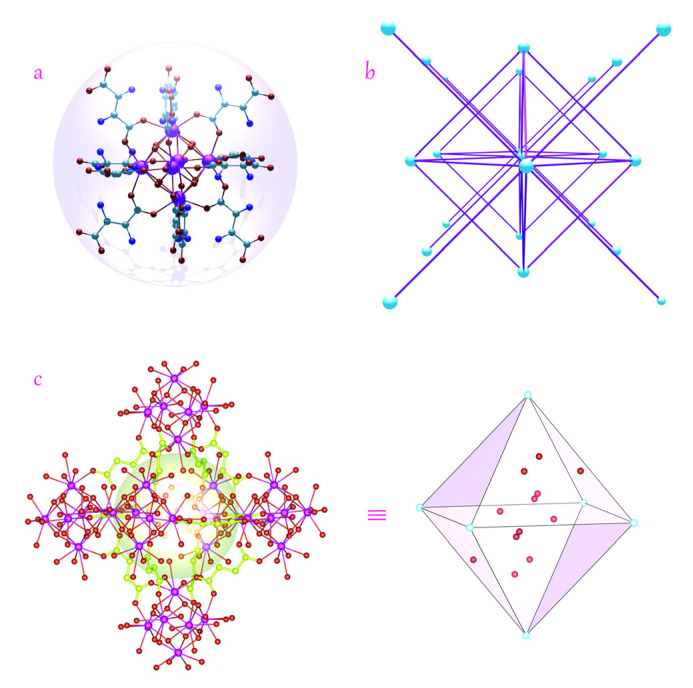
Crystal structure of **Ce-asp**: (**a**) The {Ce_6_O_14_(ASP)_12_} cluster. Ball: C, gray; O, red; N, blue; Ce, pink. (**b**) Topological representation of the *fcu* net along the *c*-axis. White ball: 12-c node. (**c**) A single octahedral cage with gold balls representing the pore space; the guest water molecules within the cage are highlighted. Some atoms are omitted for clarity.

Since the crystal structure of MOF **Ce-asp** was derived and described in detail by Norbert et al. [38], using Rietveld methods based on earlier PXRD data, we will only introduce its main structural characteristics here. Like UiO-66, this MOF contains [Ce_6_(µ_3_ − O)_4_(µ_3_ − OH)_4_(H_2_O)_6_] clusters (Figure 1a), being further bridged by 12 DL-asp^2−^ ligands to constitute a 3D framework showing an fcu topology (Figure 1b,c).

**Figure 2:**
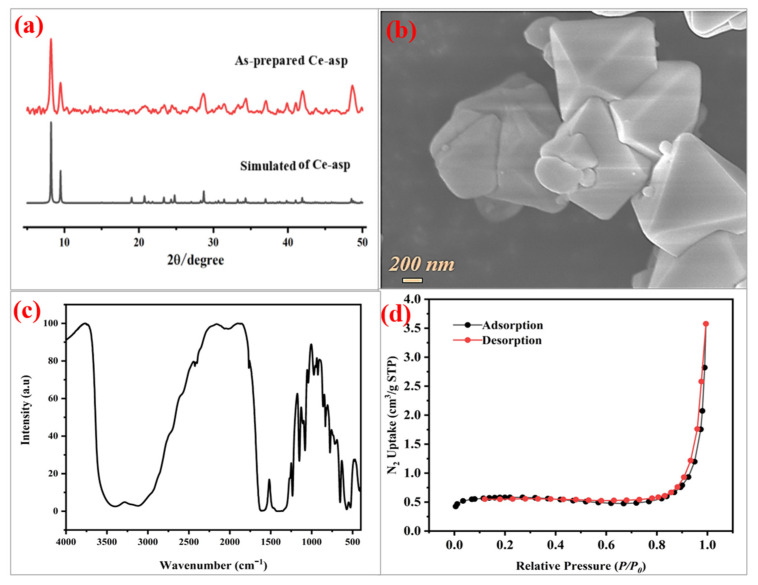
(**a**) PXRD patterns of **Ce-asp**. (**b**) SEM image of **Ce-asp**. (**c**) IR spectrum of **Ce-asp**. (**d**) N_2_ adsorption/desorption (77 K) isotherms of **Ce-asp**.

Crystallization occurs rapidly because of the strong interaction of tetravalent metal ions and carboxylic acid ligands; however, single-crystal products are often difficult to obtain, while microcrystalline powder samples are readily available. Fortunately, PXRD is a powerful technology that allows us to analyze powder items to identify whether they were properly prepared. As indicated in Figure 2a, the position and intensity of the theoretical PXRD patterns of MOF **Ce-asp** are consistent with those of the synthesized sample, which proves the successful synthesis of the MOF. Notably, the quantitative results for crystallinity could be obtained using the Jade 6.0 program by using the tested PXRD data. As manifested in Figure S2, the crystallinity of **Ce-asp** is 98.4%, indicating a high crystallinity. SEM measurements allow for a more intuitive understanding of the MOF’s crystallinity and morphology. As denoted in Figure 2b, **Ce-asp** has a regular octahedral shape and the particle sizes are in the range of 1.6–2 μm.

Figure 2c exhibits the infrared spectrum of the MOF, demonstrating that the MOF’s characteristic infrared absorption is consistent with prior observations in the literature [38]. The strong and broad vibrations around 3400 and 3205 cm^−1^ could be ascribed to the *ν*_as_(OH) and *ν*_as_(NH_2_) vibrations, respectively. The absorption peak at 1613 cm^−1^ is caused by the in-plane vibration of the N-H bond. The broad and strong peak in the range of 1560–1410 cm^−1^ can be attributed to the *ν*_as_(COO) and *ν*_s_(COO) vibrations.

Further, at 77 K, we performed nitrogen adsorption/desorption isotherm measurements on the activated MOF sample. The MOF exhibited a type-II isotherm, which is close to the previous descriptions [38]. As demonstrated in Figure 2d and Figure S3, the calculated BET-specific surface area is only 2.2 m^2^/g, showing a non-porous framework. This could be attributed to the use of the highly hydrophilic ligand DL-aspartic acid in the preparation of **Ce-asp**, which boosts the coordination number of cerium(IV) from 8 to 9, and the small DL-aspartic acid leads to a short distance between Ce-O clusters, which ultimately limits the porosity of the MOF [38]. In addition, we adopted density functional theory (DFT) to assess the pore size of the MOF, and the obtained main pore size is 1.7 nm (Figure S4).

### 3.2. Structural Stability of **Ce-Asp**

#### 3.2.1. Thermal Stability

As displayed in Figure S5, the thermogravimetric curve of MOF **Ce-asp** in an air atmosphere is mainly divided into two steps. One is between room temperature and 271 °C, corresponding to the loss of solvent molecules adsorbed within the framework. Second, as the heating temperature is above 271 °C, the organic components of the framework experience thermal decomposition, resulting in the collapse of the framework. The final residue is presumed to be CeO_2_, which is also consistent with what has been reported in the literature. In summary, the high thermal stability demonstrated by this compound is fully suitable for future applications.

#### 3.2.2. Water and Acid–Base Stability

**Figure 3:**
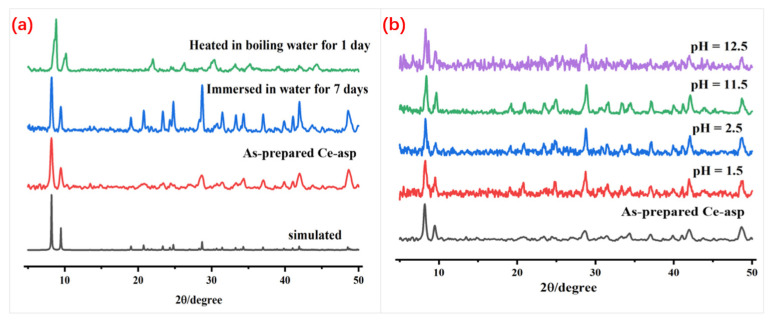
PXRD curves of **Ce-asp**: (**a**) solids after water treatment; (**b**) solids after chemical treatment.

Because this study focused on MOF **Ce-asp**’s adsorption performance for hexavalent chromium ions in aqueous solution, its water and acid–base stability were especially essential. Figure 3a compares the PXRD patterns of MOF **Ce-asp** soaked in room temperature water for a week or heated in boiling water for 24 h to the original sample’s PXRD patterns. The figure manifests that the PXRD diffraction peaks after immersion in room temperature water or reflux in boiling water are nearly identical to those before water treatment, suggesting its great water stability. Moreover, as denoted in Figure 3b, after one day of soaking in HCl and NaOH solutions with varying pH values, the PXRD patterns of **Ce-asp** are nearly identical to those of the original sample, demonstrating that **Ce-asp** can exist stably in both acidic and alkaline aqueous conditions and maintains structural inertia in the pH range of 1.5 to 12.5. At the same time, the residual weight percentages (Figure S6) of H_2_O acid–base-treated solids for **Ce-asp** were weighted and calculated to be 93.5%, 90.2%, and 91.8%, respectively, demonstrating the MOF’s great stability. The high structural stability of this Ce(IV)-MOF is very close to that of similar Ce-based MOFs reported in the literature [39,40,41,42,43,44], showing the structural advantages of the cluster Ce(IV)-MOFs.

### 3.3. Cr(IV) Adsorption Properties of **Ce-Asp**

The preceding studies demonstrated that the MOF has strong structural stability, particularly in terms of water and chemical stability, laying the groundwork for investigating hexavalent chromium ion adsorption in aqueous solution. As a result, we will choose a certain concentration of hexavalent chromium ion aqueous solution to investigate the effects of pH, adsorption period, and initial MOF dosage on MOF adsorption performance to achieve the optimal adsorption conditions.

#### 3.3.1. Effect of pH on Adsorption Properties of **Ce-Asp**

At room temperature, eight sections of 20 mg of **Ce-asp** were placed in 10 mL of 100 mg/L Cr^6+^ solution, respectively. The pH values of the solutions were tuned by NaOH or HCl, respectively, to a value of 1, 2, 3, 4, 5, 6, 8, or 10, and then agitated for 8 h. Following centrifugation, each solution’s absorbance at 350 nm after adsorption was determined (Figure S7). The absorbance working curve was used to calculate the Cr(VI) content in each solution, as well as the rate of Cr(VI) removal by the MOF (Table S1). As exhibited in Figure S7 and Table S1, As the pH value increased, this MOF’s adsorption ability for Cr(VI) declined. The maximum adsorption capacity and clearance rate of the MOF were found at pH = 1. This could be because, at pH = 1, Cr(VI) ions primarily exist in the forms of HCr_2_O_7_^−^ and HCrO_4_^−^. H^+^ ions in water can combine with the NH_2_ groups on the surface of the MOF to form an NH_3_^+^ group, and the electrostatic attraction between the positively charged group and the negatively charged Cr(VI) leads to Cr(VI) adsorption. As the pH values of the solution increase, the positively charged groups on the surface of the complex lose H^+^, become neutral groups, and cannot attract Cr_2_O_7_^2−^ ions well in the solution by electrostatic attraction [45].

#### 3.3.2. Effect of Time on Adsorption Properties of **Ce-Asp**

**Figure 4:**
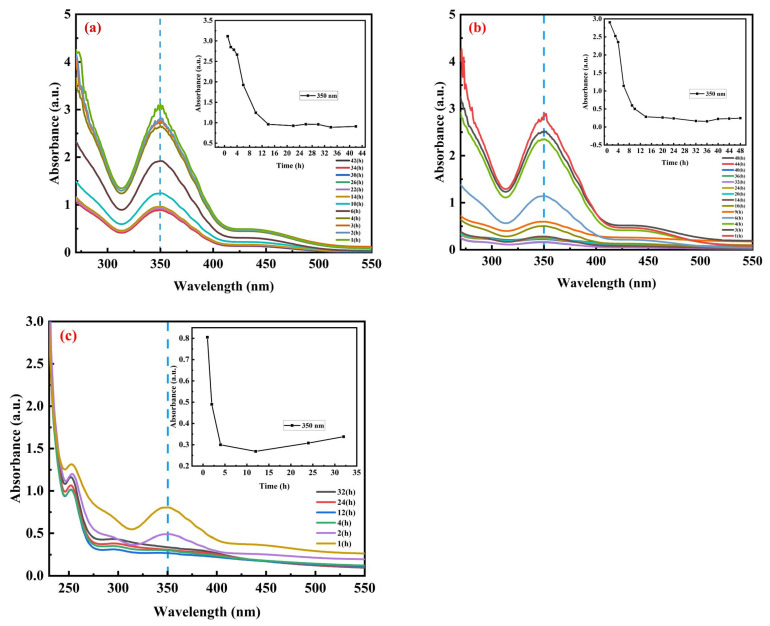
Influence of adsorption time of MOF on absorbance curve of hexavalent chromium: (**a**) MOF dosage of 10 mg; (**b**) MOF dosage of 20 mg; and (**c**) MOF dosage of 30 mg.

The 10 mg, 20 mg, and 30 mg MOF samples were, respectively, put into glass bottles containing 10 mL of Cr(VI) solution (100 mg/L). Under the condition of 25 °C, an adsorption experiment was performed for 1–48 h, and the measured absorbance curve is shown in Figure 4. The calculated adsorption capacity and removal rate are shown in Tables S2–S4. It can be seen from the data in Tables S2–S4 that with the increasing adsorption time, the adsorption amount of the MOF also continues to increase, but its trend is gradual and finally reaches saturation. The adsorption rate of 1–4 h is fast, and then the adsorption rate slows down. When the dosage of the MOF is 10, 20, and 30 mg, it takes 14 h to reach the adsorption equilibrium. The adsorption rate of the MOF decreased significantly from 14 h to 48 h.

#### 3.3.3. Effect of Initial Dosage of the MOF on Adsorption Properties

**Figure 5:**
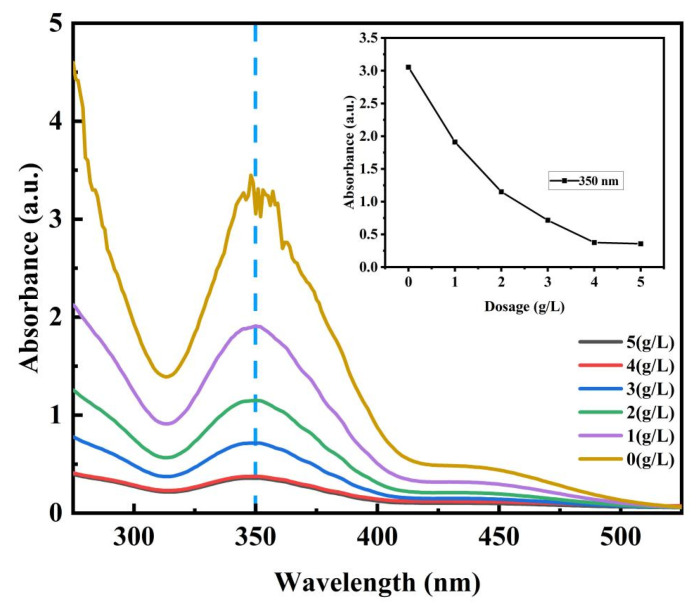
Effect of MOF dosage on absorbance curve of hexavalent chromium.

The 10, 20, 30, 40, and 50 mg MOFs were put into 10 mL glass bottles of 100 g/L Cr^6+^ solution, respectively. After adsorption at 25 °C for 5 h, the measured absorbance curve is shown in Figure 5, and the calculated adsorption capacity and removal rate are shown in Table S5. With the boost in the dosage of the MOF, the adsorption capacity decreases, and the removal rate increases. With the increasing MOF dosage, the *R* of Cr(VI) showed a trend of increasing at first and then flattening. Owing to the increasing dosage of the MOF, more adsorption sites were generated and the probability of collision with Cr(VI) in the solution was increased. Finally, Cr(VI) ions in the solution reached the adsorption equilibrium, and the final *R* was 90%. According to the R values, the MOF dosage of 30 mg for 10 mL of Cr(VI) solution is appropriate.

#### 3.3.4. Effect of Initial Solution Concentration on Adsorption Properties of the MOF

An amount of 10 mg of the MOF was injected into 10 mL of Cr^6+^ solution of 20, 40, 60, 80, and 100 mg/L, respectively. Under the condition of 25 °C and adsorption for 6 h, the measured absorbance curve is shown in Figure S8, and the calculated *Q*_e_ and *R* are shown in Table S6. With the increasing initial solution concentration, the adsorption capacity increases and the removal rate decreases. As the concentration of Cr(VI) soared from 20 to 100 mg/L, the *Q*_e_ of the MOF showed a trend of increasing. The reason is that the mass of the complex is certain and the active adsorption site is also certain, and with the rising Cr(VI) concentrations, more Cr(VI) will be transferred from the solution to the surface of the MOF, leading to a gradual increase in *Q*_e_.

#### 3.3.5. Study on Adsorption Mechanism of MOF

1. Adsorption kinetics exploration

The diagrams of the quasi-first-order and quasi-second-order models are shown in Figure S9a and S9b, respectively. As denoted in Table S7, the *R*_2_ of the quasi-second-order kinetic model is 0.9909, larger than that of the quasi-first-order model (0.9574), indicating that the former model could preferably describe the Cr(VI) adsorption behavior of **Ce-asp**. The theoretical *Q*_e_ obtained by this model is also consistent with the experimental *Q*_e_, which demonstrates that the adsorption behavior is mostly chemical adsorption.

2. Adsorption isotherm exploration

The adsorption data of the MOF were fitted by isotherm models, which were Langmuir adsorption isotherm and Freundlich adsorption isotherm models. The fitting results are shown in Figure S10a and S10b, respectively. As listed in Table S8, for the adsorption of Cr(VI), the *R*_2_ of the Langmuir isotherm model at 26 °C is 0.975, which is higher than that of the Freundlich model (0.876), indicating that the Langmuir model can better depict the adsorption process of Cr(VI), and the adsorption is monolayer adsorption.

#### 3.3.6. Reuse Experiment of MOF

An amount of 40 mg of **Ce-asp** was placed in 10 mL of Cr(VI) solution (100 mg/L) for a repeatability test. The adsorption time was set to 0 to 14 h. Then, the MOF adsorbed to dynamic equilibrium was placed in 0.01 mol/L sodium hydroxide solution at 25 °C and oscillated for 0.5 h to desorb the chromium ions. Then, it was cleaned with deionized water until neutral, and then the dried MOF was reused in the adsorption–desorption experiment, which was repeated four times. The removal rate was calculated by comparing the adsorption amount of the four adsorption experiments with that of the first adsorption experiment.

As displayed in Figure S11 and Table S9, with the increase in the number of repeated adsorption and desorption, the removal rate of the titular MOF for hexavalent chromium ions decreased significantly, from 99.8% to 20%, but in the first three repeated tests, the lowest removal rate still reached more than 45%, showing a good application potential.

To investigate whether the concentration of Cr(VI) in the test solution treated with **Ce-asp** adsorbent met the WHO standard that the maximum concentration of Cr(VI) in drinking water does not exceed 0.05 mg/L, we conducted an additional experiment. Note that in this research, we primarily used the self-color development of bichromate to quantify absorbance. When the concentration of Cr(VI) in the solution is extremely low, it is impossible to measure absorbance. As a result, we added the color-developing agent dibenzoyl dihydrazine to the MOF adsorbent-treated solution to determine the low concentration of Cr(VI) (the method is according to the GB 7467-87) [46]. Following a series of experiments, it was discovered that, assuming that the MOF dose is adequate and the adsorption duration is sufficient, the concentration of Cr(VI) in the treated solution is less than 0.05 mg/L, meeting the WHO drinking water standard.

In general, based on the above test results, if both the adsorption rate of the MOF (74.1 mg/g) and the removal rate (74.1%) of Cr(VI) are considered, the best experimental conditions are in a 10 mL of potassium dichromate solution (100 mg/L), with a MOF dosage of 10 mg and an adsorption time of 34 h. If only the removal rate of the MOF for Cr (VI) is considered, then the best experimental conditions are that the dosage of the MOF is 20 mg in 10 mL of potassium dichromate solution (100 mg/L) and the adsorption time is set to 36 h.

Finally, by comparing the adsorption rates of the titular MOF to those of COFs, MOFs, and related materials reported in the literature (Table S10), it is discovered that, while the performance of the titular MOF is lower than that of the newly emerged crystalline material COFs [47,48,49,50,51], it is still superior to the majority of the reported MOFs [52,53,54,55,56,57,58,59,60,61]. More importantly, **Ce-asp** employs inexpensive natural amino acids as bridging ligands, demonstrating cost advantages in future practical applications.

## 4. Conclusions

To summarize, we used water as a quick reaction solvent and a cheap amino acid as a bridging ligand to create a highly structurally stable three-dimensional cerium-based MOF equivalent to UiO-66. The adsorption characteristics of Cr(VI) ions in aqueous solution were thoroughly investigated. The results demonstrate that it has good hexavalent chromium ion adsorption capabilities. Under optimal experimental conditions, the adsorption and removal rates can be as high as 74.1 mg/g and 96%, indicating a high application value. The adsorption mechanism was investigated further, and it was determined to be monolayer chemisorption.

## Data Availability

The original contributions presented in the study are included in the article and Supplementary Materials, further inquiries can be directed to the corresponding authors.

## Supplementary Materials

The following supporting information can be downloaded at: https://www.mdpi.com/article/10.3390/ma17215293/s1, Figure S1: Working curve of the concentration of hexavalent chromium on absorbance in aqueous solution; Figure S2: The calculated crystallinity of **Ce-asp**; Figure S3: The BET surface area plot of **Ce-asp**; Figure S4: Differential pore volume vs. pore width of **Ce-asp**; Figure S5: TG curve of **Ce-asp**; Figure S6: Percentage of **Ce-asp** remaining after water, acid and base treatment; Figure S7: Effect of pH on absorbance the solutions upon MOF adsorption Cr(VI); Figure S8: Effect of initial solution concentration on absorbance curve of MOF adsorbed hexavalent chromium; Figure S9: Adsorption kinetics fit curve (a) quasi-first-order dynamic model, (b) quasi-second-order dynamic model; Figure S10: Adsorption isotherm fitting curves of MOF (a) Langmuir adsorption isotherm model, (b) Freundlich adsorption isotherm model; Figure S11: Plot of absorbance data obtained from repeated adsorption–desorption experiments for **Ce-asp**; Table S1: Adsorption data of the MOF for chromium(VI) at different pH values; Table S2: Data of adsorption properties of MOF (10 mg) for hexavalent chromium at different adsorption times; Table S3: Data of adsorption properties of MOF (20 mg) for hexavalent chromium at different adsorption times; Table S4: Data of adsorption properties of MOF (30 mg) for hexavalent chromium at different adsorption times; Table S5: Adsorption properties of MOF for hexavalent chromium at different dosage; Table S6: Data of adsorption properties of MOF to Cr(VI) at different initial solution concentrations; Table S7: Adsorption related kinetic model parameters of the MOF; Table S8: Adsorption isotherm parameters of the MOF; Table S9: Repeated experimental data of adsorption and desorption for the MOF; Table S10: Comparison of adsorption properties of Cr(VI) of **Ce-asp** and related crystalline materials (MOFs, COFs, etc.) in the literature [46,47,48,49,50,51,52,53,54,55,56,57,58,59,60].
