# Supplementary material for: Adsorption Performance for Chromium(VI) of a UiO-66-Ce Metal–Organic Framework Built by DL-Aspartic Acid
Source: Materials (Basel). 2024 Oct 30;17(21):5293. doi: 10.3390/ma17215293 (PMC11547581; doi:10.3390/ma17215293)
Supplement: Supplementary file 1 [file materials-17-05293-s001.zip › materials-3286409-supplementary.pdf]

Supporting information

Adsorption Performance for Chromium(VI) of a UiO-66-Ce Metal-Organic Framework Built by DL-Aspartic Acid

Xiao-Yi Lin[1], Sabrina Yanan Jiang[1,*] and Gang Li[2,*]

[1] National Observation and Research Station of Coastal Ecological Environments in Macao, Macao Environmental Research Institute, Macau University of Science and Technology, Taipa 999078, Macao, China; 2230028761@must.edu.mo
[2] College of Chemistry, Zhengzhou University, Zhengzhou 450001, Henan, China
* Correspondence: ynjiang@must.edu.mo (S.Y.J.); gangli@zzu.edu.cn (G. L.)

Number of pages: 12
Number of Figures:11
Number of Tables:10

List

Figure S1: Working curve of the concentration of hexavalent chromium on absorbance in aqueous solution;
Figure S2: The calculated crystallinity of Ce-asp.
Figure S3: The BET surface area plot of Ce-asp;
Figure S4: Differential pore volume vs. pore width of Ce-asp;
Figure S5: TG curve of Ce-asp;
Figure S6: Percentage of Ce-asp remaining after water, acid and base treatment;
Figure S7: Effect of pH on absorbance the solutions upon MOF adsorption Cr(VI);
Figure S8: Effect of initial solution concentration on absorbance curve of MOF adsorbed hexavalent chromium;
Figure S9: Adsorption kinetics fit curve (a) quasi-first-order dynamic model, (b) quasi-second-order dynamic model;
Figure S10: Adsorption isotherm fitting curves of MOF (a) Langmuir adsorption isotherm model, (b) Freundlich adsorption isotherm model;
Figure S11: Plot of absorbance data obtained from repeated adsorption-desorption experiments for Ce-asp;
Table S1: Adsorption data of the MOF for chromium(VI) at different pH values;
Table S2: Data of adsorption properties of MOF (10 mg) for hexavalent chromium at different adsorption times;
Table S3: Data of adsorption properties of MOF (20 mg) for hexavalent chromium at different adsorption times;
Table S4: Data of adsorption properties of MOF (30 mg) for hexavalent chromium at different adsorption times;
Table S5: Adsorption properties of MOF for hexavalent chromium at different dosage;
Table S6: Data of adsorption properties of MOF to Cr(VI) at different initial solution concentrations;
Table S7: Adsorption related kinetic model parameters of the MOF;
Table S8: Adsorption isotherm parameters of the MOF.
Table S9. Repeated experimental data of adsorption and desorption for the MOF.
Table S10. Comparison of adsorption properties of Cr(VI) of Ce-asp and related crystalline materials (MOFs, COFs, etc.) in the literature.

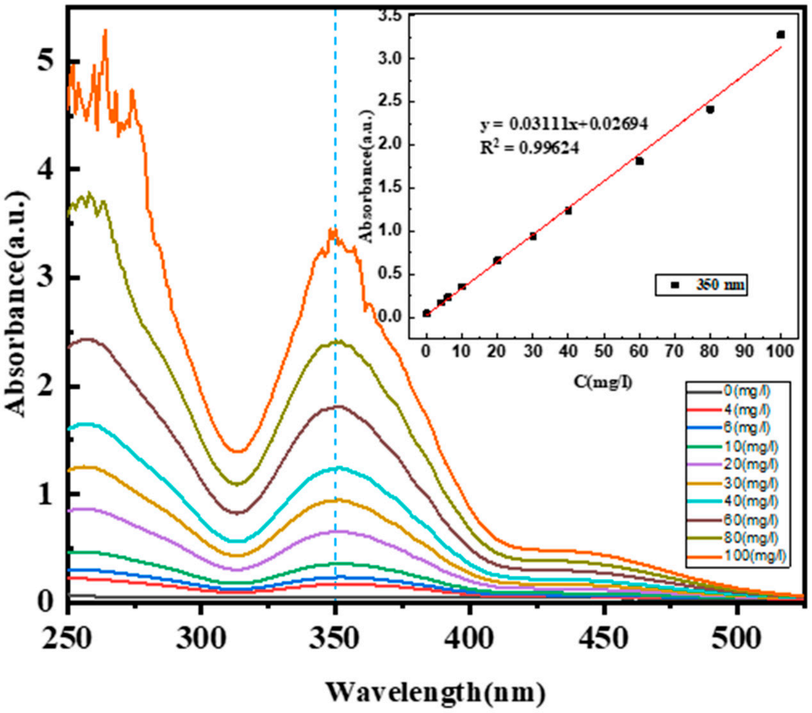

Figure S1. Working curve of the concentration of hexavalent chromium on absorbance in aqueous solution.

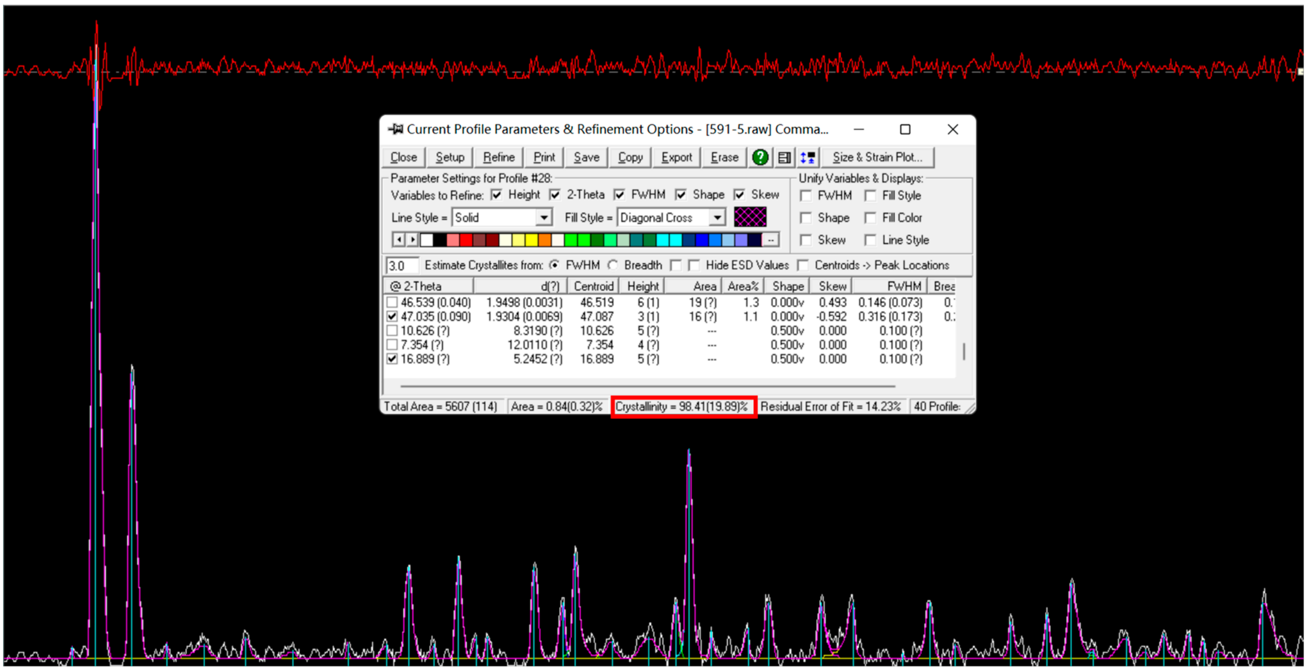

Figure S2. The calculated crystallinity of **Ce-asp**.

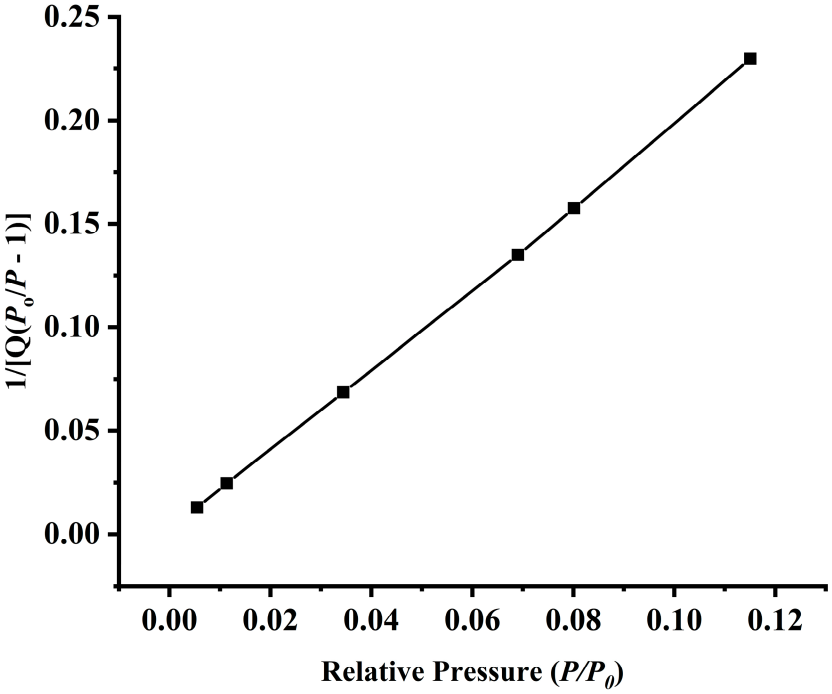

Figure S3. The BET surface area plot of **Ce-asp**.

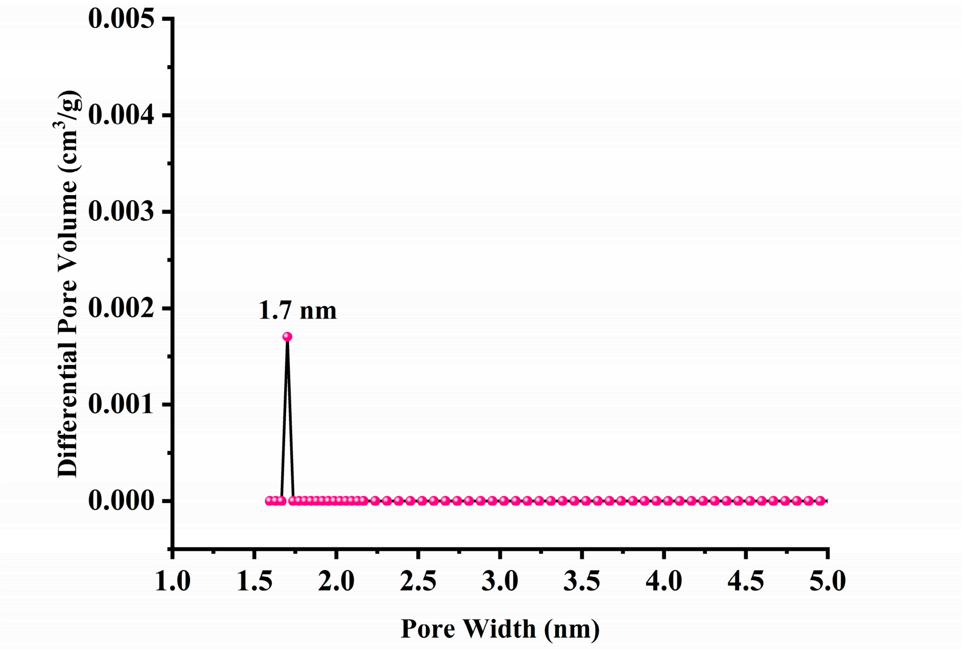

Figure S4. Differential pore volume *vs*. pore width of **Ce-asp**.

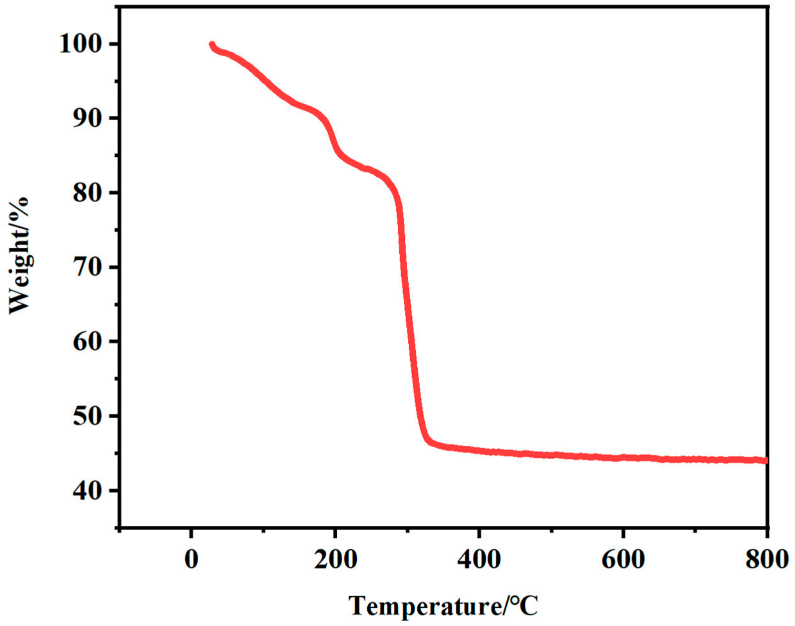

Figure S5. TG curve of **Ce-asp**.

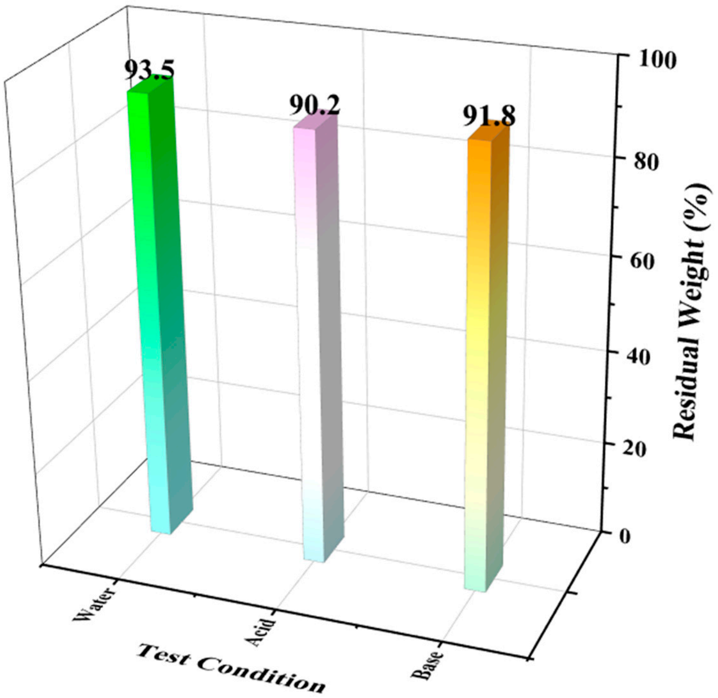

Figure S6. Percentage of **Ce-asp** remaining after water, acid and base treatment.

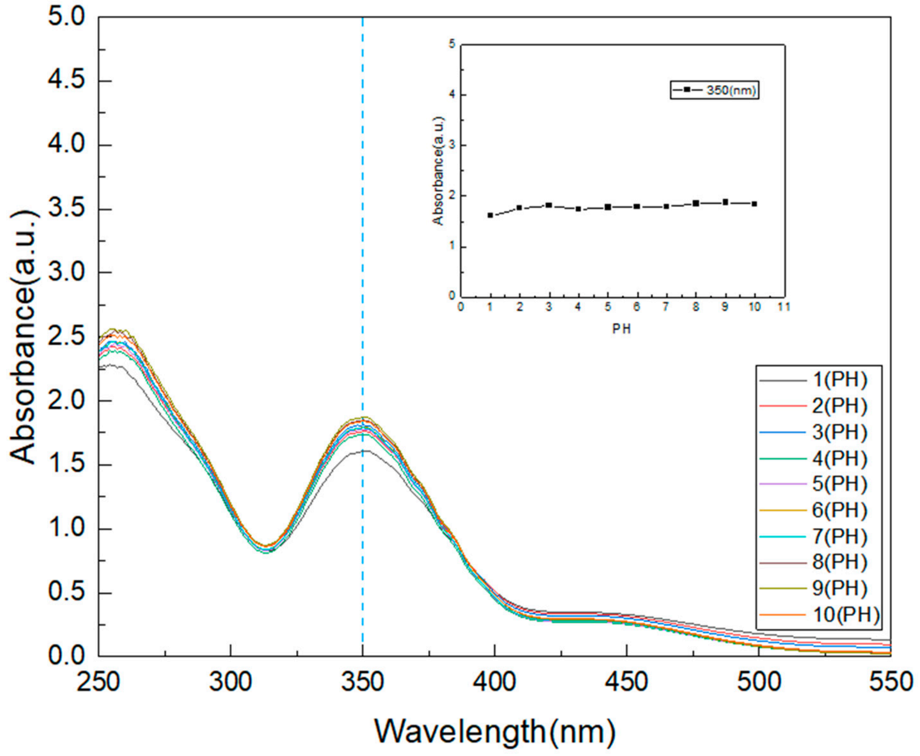

Figure S7. Effect of pH on absorbance the solutions upon MOF adsorption Cr(VI).

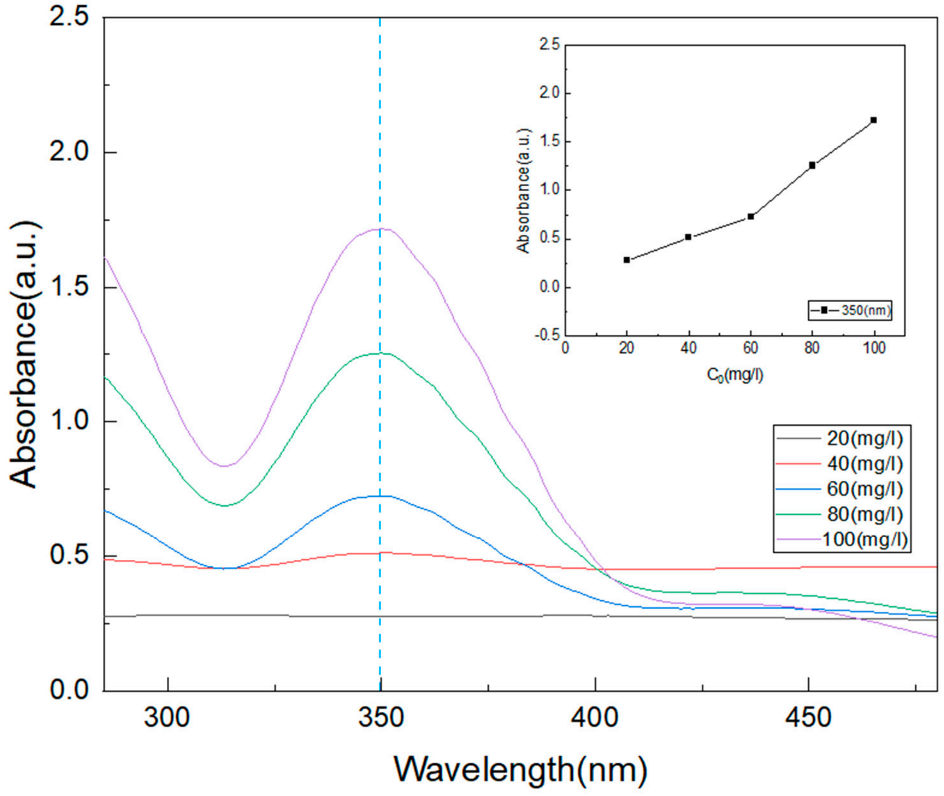

Figure S8. Effect of initial solution concentration on absorbance curve of MOF adsorbed hexavalent chromium.

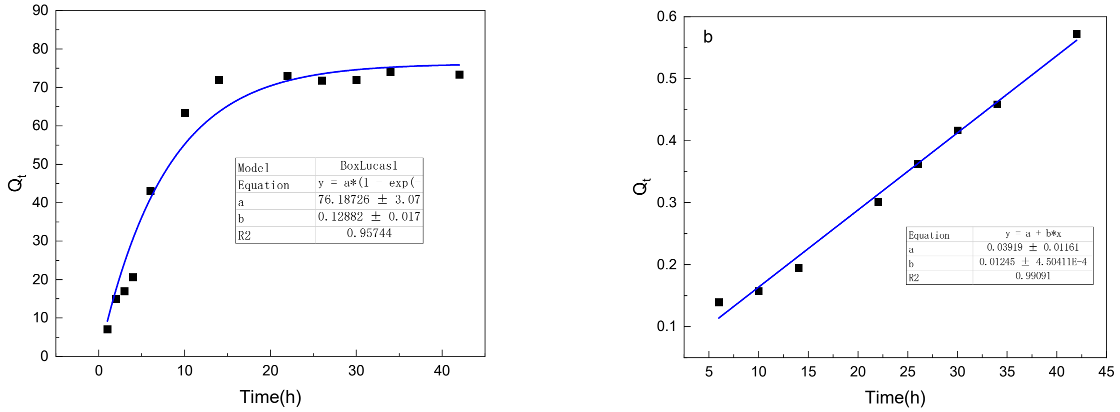

(a) (b)

Figure S9. Adsorption kinetics fit curve (a) quasi-first-order dynamic model, (b) quasi-second-order dynamic model.

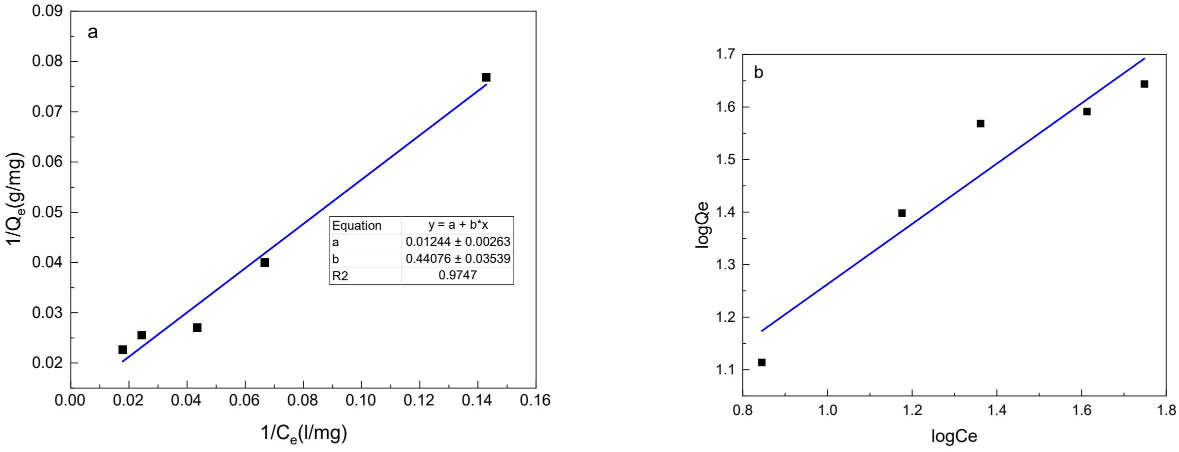

(a) (b)

Figure S10. Adsorption isotherm fitting curves of MOF (a) Langmuir adsorption isotherm model, (b)Freundlich adsorption isotherm model.

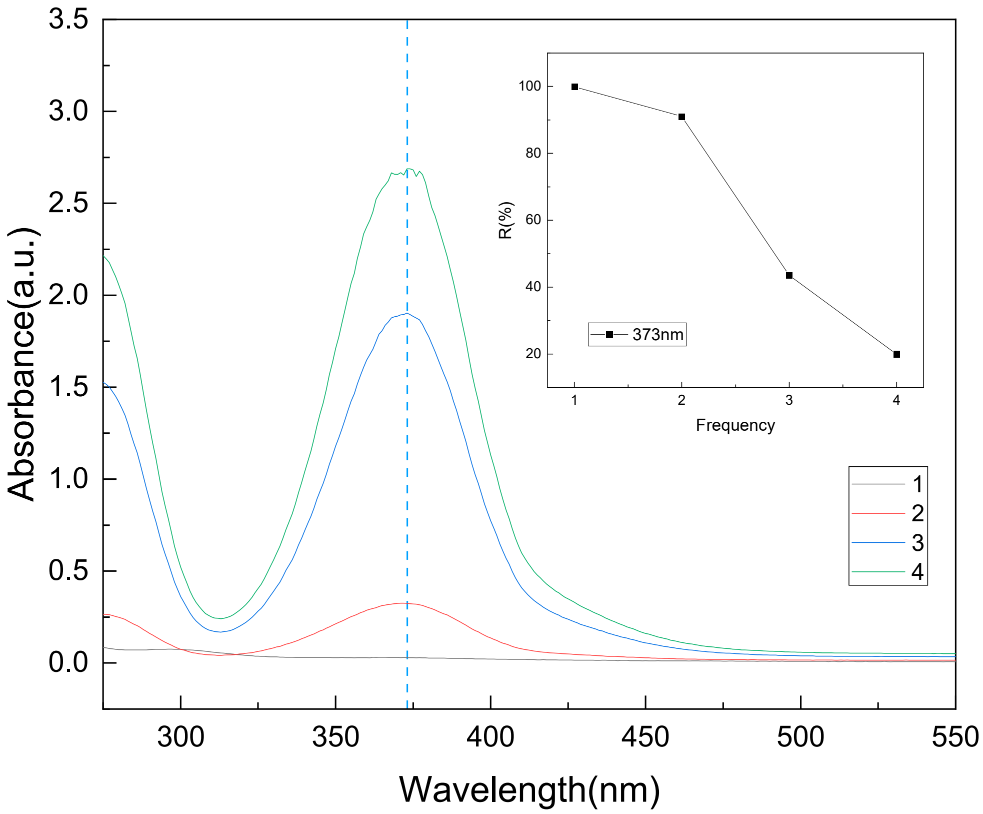

Figure S11. Plot of absorbance data obtained from repeated adsorption-desorption experiments for **Ce-asp**.

Table S1. Adsorption data of the MOF for chromium(VI) at different pH values.

pH values	1	2	3	4	5	6	7	8	9	10
Abs	1.609	1.761	1.812	1.74	1.778	1.794	1.791	1.853	1.877	1.845
C_1(mg/l)	53	58	60	57	58	59	59	61	62	61
Q_t（mg/g）	24	21	20	21	21	21	21	20	19	20
R	47%	42%	40%	43%	42%	41%	41%	39%	38%	39%

Table S2. Data of adsorption properties of MOF (10 mg) for hexavalent chromium at different adsorption times

Time(h)	1	2	3	4	6	10	14	22	26	30	34	42
Abs	3.112	2.849	2.783	2.663	1.922	1.243	0.961	0.926	0.962	0.959	0.888	0.91
C_1(mg/l)	92.9	85.0	83.0	79.4	57.1	36.6	28.1	27.1	28.2	28.1	25.9	26.6
Q_e (mg/g)	7.15	15.0	17.0352	20.6	42.9	63.4	71.9	72.9	71.8	71.9	74.1	73.4
R	7.1%	15.1%	17.0%	20.7%	43.0%	63.4%	71.9%	72.9%	71.8%	71.9%	74.1%	73.4%

Table S3. Data of adsorption properties of MOF (20 mg) for hexavalent chromium at different adsorption times

Time(h)	1	3	4	6	9	10	14	20	24	32	36	40	44	48
Abs	2.902	2.524	2.354	1.142	0.598	0.503	0.283	0.263	0.238	0.167	0.158	0.224	0.233	0.246
C_l(mg/l)	86.5	75.2	70.1	33.6	17.2	14.4	7.7	7.1	6. 4	4.2	4.0	6.0	6.29	6.69
Q_e（mg/g	6.7	12.4	15.0	33.2	41.4	42.8	46.1	46.4	46.8	47.9	48.0	47.0	46.9	46.7
R	13.5%	24.8%	30.0%	66.4%	82.8%	85.7%	92.3%	92.9%	93.6%	95.8%	96.0%	94.0%	93.8%	93.4%

Table S4. Data of adsorption properties of MOF (30 mg) for hexavalent chromium at different adsorption times

Time(h)	1	2	4	12	24	32
Abs	0.805	0.49	0.3	0.269	0.308	0.338
C_l(mg/l)	23.4	13.9	8.2	7.3	8.5	9.4
Q_e（mg/g)	25.5	28.7	30.6	30.9	30.5	30.2
R	76. 6%	86.0%	91.8%	92.7%	91.5%	90.6%

Table S5. Adsorption properties of MOF for hexavalent chromium at different dosage

Dosage(g/l)	0	1	2	3	4	5
Abs	3.054	1.911	1.151	0.761	0.376	0.358
C_e(mg/l)	100	63	37	22	11	10
Q_t（mg/g）	0	37	31.5	26	22.25	18
R	0	37%	63%	88%	89%	90%

Table S6. Data of adsorption properties of MOF to Cr(VI) at different initial solution concentrations

C_0(mg/l)	20	40	60	80	100
Abs	0.276	0.512	0.724	1.256	1.718
C_e(mg/l)	7	15	23	41	56
Q_e（mg/g)	13	25	37	39	44
R	65%	63%	62%	49%	44%

Table S7. Adsorption related kinetic model parameters of the MOF

Cr(VI)'s concentration	Quasi-first-order dynamic model			quasi-second-order dynamic model		
mg/L	k_1 (h^{-1})	Q_e (mg/g)	R^2	k_2 (h·g/mg)	Q_e (mg/g)	R^2
100	0.12882	76.18726	0.95744	0.00396	80.32129	0.99091

Table S8. Adsorption isotherm parameters of the MOF

Temperature	Langmuir adsorption isotherm model			Freundlich adsorption isotherm model		
℃	Q_m (mg/g)	k_3	R^2	n	k_4($mg^{(1-n)}\cdot L^n\cdot g^{-1}$)	R^2
26	80.3859	0.02822	0.9747	1.74283	2.75905	0.87645

Table S9. Repeated experimental data of adsorption and desorption for the MOF

Number of regeneration	1	2	3	4
Abs	0.029	0.324	1.903	2.688
C_1(mg/l)	0.09	8.97	56.48	80.11
Q_e (mg/g)	24.98	22.76	10.88	4.97
R	100%	91%	45%	20%

Table S10. Comparison of adsorption properties of Cr(VI) of **Ce-asp** and related crystalline materials (MOFs, COFs, etc.) in the literature

Materials	Adsorption Quantity (mg/g)	Concentration (mg/L)	Ref.
COF2	649	5	[1]
COF1	463	5	[1]
PGMA-PEI60	461	100	[2]
COF BTA-DHBZ	384	-	[3]
COF Tp_DGCl	360	10	[4]
CON-1	293	-	[5]
$[Cd(tipo)(HCOO)(H_2O)]NO_3 \cdot DMF$	228	600	[6]
BUT-39	215	0.79	[7]
Zr-MSA	202	28	[8]
$[\{Ni_2(L)_3(SO_4)(H_2O)_3\}SO_4 \cdot xG]_n$	166	-	[9]
UiO-66	164	100	[10]
MOF-801	156.2	1000	[11]
TMU-30	145	5.5	[12]
ZIF-8	112	50	[13]
NiCo-LDH	99.9	62	[14]
UiO-66	86	50	[13]
**Ce-asp**	74.1	100	This work
MIL-100(Fe)	74	100	[15]
FIR-53	74	312	[16]
PCN-134	73.5	-	[17]
Dy-MOF	63	-	[18]
MOF-5	62	40	[19]
SLUG-21	60	-	[20]
UPC-50	56.8	120	[21]
MOF-867	54	400	[22]
BU-100	49	50	[23]
Cu-BTC	48	20	[24]
MIL-100(Fe)-Na_2CO_3	46.02	145	[25]
ZIF-8@CA	41.8	-	[26]
MIL-101(Cr)	40	100	[27]

UiO-66-NH_2	32.36	80	[28]
ZIF-8	25	20	[29]
UiO-67	20	50	[23]
ZIF-67	15	30	[30]
PCN-224-MMA(O)	5.4	-	[31]
HKUST-1	3	50	[23]
ZIF-8	<1	20	[23]

References

1. Donghai, Z.; Shuangxi, Z.; Ziming, Z.; Rui, L.; Jia, Y.; Xu, Z.; Shenjie, L.; Yunhai, Z.; Shiyu, M.; Wei, W. Highly efficient and selective removal of cr(vi) by covalent organic frameworks: structure, performance and mechanism. *Colloids and Surfaces a: Physicochemical and Engineering Aspects* **2020**, *600*, 124910-124919.
2. Xitong, S.; Liangrong, Y.; Huifang, X.; Junmei, Z.; Xiaopei, L.; Yinbin, H.; Huizhou, L. High capacity adsorption of cr(vi) from aqueous solution using polyethylenimine-functionalized poly(glycidyl methacrylate) microspheres. *Colloids and Surfaces a: Physicochemical and Engineering Aspects* **2014**, *457*, 160-168.
3. Fu-Zhi, C.; Rong-Ran, L.; Qiao-Yan, Q.; Guo-Fang, J.; Xin, Z. Efficient removal of cr(vi) from aqueous solutions by a dual-pore covalent organic framework. *Adv. Sustain. Syst.* **2019**, *3*, 1800150-1800156.
4. Zhuang, X.; Hao, J.; Zheng, X.; Fu, D.; Mo, P.; Jin, Y.; Chen, P.; Liu, H.; Liu, G.; Lv, W. High-performance adsorption of chromate by hydrazone-linked guanidinium-based ionic covalent organic frameworks: Selective ion exchange. *Sep. Purif. Technol*. **2021**, *274*, 118993.
5. Wu, W.; Li, F.; Yao, B.; Ding, L.; Kan, J.; Liu, F.; Zhao, G.; Wang, S.; Dong, Y. Synthesis of covalent organic frameworks via kabachnik-fields reaction for water treatment. *J. Hazard. Mater.* **2022**, *433*, 128831.
6. Guo, M.; Guo, H.; Liu, S.; Sun, Y.; Huo, X. A microporous cationic metal–organic framework for the efficient removal of dichromate and the selective adsorption of dyes from water. *RSC Adv*. **2017**, *7*, 51021-51026.
7. He, T.; Zhang, Y.; Kong, X.; Yu, J.; Lv, X.; Wu, Y.; Guo, Z.; Li, J. Zr(iv)-based metal-organic framework with t-shaped ligand: unique structure, high stability, selective detection, and rapid adsorption of cr2o72- in water. *Acs Appl. Mater. Interfaces* **2018**, *10*, 16650-16659.
8. Yang, P.; Shu, Y.; Zhuang, Q.; Li, Y.; Gu, J. Metal-organic frameworks bearing dense alkyl thiol for the efficient degradation and concomitant removal of toxic cr(vi). *Langmuir* **2019**, *35*, 16226-16233.
9. Desai, A.V.; Manna, B.; Karmakar, A.; Sahu, A.; Ghosh, S.K. A water-stable cationic metal-organic framework as a dual adsorbent of oxoanion pollutants. *Angew. Chem. Int. Ed.* **2016**, *55*, 7811-7815.
10. Jiang, M.; Fu, W.; Wang, Y.; Xu, D.; Wang, S. Machine-learning-driven discovery of metal–organic framework adsorbents for hexavalent chromium removal from aqueous environments. *J. Colloid. Interf. Sci*. **2024**, *662*, 836-845.
11. Zheng, M.; Zhao, X.; Wang, K.; She, Y.; Gao, Z. Highly efficient removal of Cr(VI) on a stable metal–organic framework based on enhanced h-bond interaction, *Ind. Eng. Chem. Res*. **2019**, *58*, 23330–23337.
12. Aboutorabi, L.; Morsali, A.; Tahmasebi, E.; Buyukgungor, O. Metal–organic framework based on isonicotinate N-oxide for fast and highly efficient aqueous phase Cr(VI) adsorption. *Inorg. Chem*. **2016**, *55*, 5507-5513.
13. Noraee, Z.; Jafari, A.; Ghaderpoori, M.; Kamarehie, B.; Ghaderpoury, A. Use of metalorganic framework to remove chromium (VI) from aqueous solutions, *J. Environ. Health Sci. Eng*. **2019**, *17*, 701–709.
14. Hu, H. J.; Liu, J. Y.; Xu, Z. H.; Zhan,g L. Y.; Cheng, B.; Ho, W. K. Hierarchical porous Ni/Co-LDH hollow dodecahedron with excellent adsorption property for Congo red and Cr(VI) ions. *Appl. Surf. Sci.* **2019**, *478*, 981-990.
15. Nasrollahpour, A.; Moradi, S.E. Hexavalent chromium removal from water by ionic liquid modified metal-organic frameworks adsorbent, *Microporous Mesoporous Mater*. **2017**, *243*, 47–55.
16. Fu, H.R.; Xu, Z.X.; Zhang, J. Water-stable metal–organic frameworks for fast and high dichromate trapping via single-crystal-to-single-crystal ion exchange, *Chem. Mater.* **2015**, *27*, 205–210.
17. Yuan, S.; Qin, J.; Zou, L.; Chen, Y.; Wang, X.; Zhang, Q.; Zhou, H. Thermodynamically guided synthesis of mixed-linker zr-mofs with enhanced tunability. *J. Am. Chem. Soc*. **2016**, *138*, 6636-6642.
18. Shi, P.F.; Zhao, B.; Xiong, G.; Hou, Y.L.; Cheng, P. Fast capture and separation of, and luminescent probe for, pollutant chromate using a multi-functional cationic heterometal-organic framework, *Chem. Commun*. **2012**, *48*, 8231–8233.
19. Babapour, M.; Hadi Dehghani, M.; Alimohammadi, M.; Moghadam Arjmand, M.; Salari, M.; Rasuli, L.; Mubarak,N.M.; Ahmad Khan, N. Adsorption of Cr(VI) from aqueous solution using mesoporous metal-organic framework-5

functionalized with the amino acids: characterization, optimization, linear and nonlinear kinetic models, *J. Mol. Liq.* 2022,*345*, 117835.
20. Fei, H.; Bresler, M.R.; Oliver, S.R.J. A new paradigm for anion trapping in high capacity and selectivity: crystal-to-crystal transformation of cationic materials, *J. Am. Chem. Soc*. **2011**, *133*, 11110–11113.
21. Zhang, X. R.; Wang, X.; Fan, W. D.; Wang, Y. T.; Wang, X. K.; Zhang, K.; Sun D. F. A multifunctional Zr-MOF for the rapid removal of $Cr_2O_7^{2-}$, efficient gas adsorption/separation, and catalytic performance. *Mater. Chem. Front.*, **2020**, *4*, 1150-1157.
22. Zhang, Q.; Yu, J.; Cai, J.; Zhang, L.; Cui, Y.; Yang, Y.; Chen, B.; Qian, G. A porous Zrcluster-based cationic metal–organic framework for highly efficient Cr2O7 2− removal from water, *Chem. Commun*. **2015**, *51*, 14732–14734.
23. Lin, Z.J.; Zheng,H.Q.; Zheng, H.Y.; Lin, L.P.; Xin,Q.; Cao, R. Efficient capture and effective sensing of Cr2O7 2– from water using a zirconium metal–organic framework, *Inorg. Chem*. **2017**, *56*, 14178–14188.
24. Maleki, A.; Hayati, B.; Naghizadeh, M.; Joo, S.W. Adsorption of hexavalent chromium by metal organic frameworks from aqueous solution, *J. Ind. Eng. Chem*. **2015**, *28*, 211–216.
25. Fang, Y.; Wen, J.; Zeng, G. M.; Jia, F. Y.; Zhang, S. Y.; Peng, Z. L.; Zhang, H. B. Effect of mineralizing agents on the adsorption performance of metal–organic framework MIL-100(Fe) towards chromium(VI). *Chem. Eng. J*. **2018**, *337*, 532-540.
26. Bo, S. G.; Ren, W. J.; Lei, C.; Xie, Y. B.; Cai, Y. R.; Wang, S. L.; Gao, J. K.; Ni, Q. Q.; Yao, J. M. Flexible and porous cellulose aerogels/zeolitic imidazolate framework (ZIF-8) hybrids for adsorption removal of Cr(IV) from water. *J. Solid State Chem*. **2018**, *262*, 135-141
27. Liu, Z.N.; Fan, A.P.; Han, X.G.; Shapour, H.; Zhang, Q.Y. PEI-modified chromiumbased metal organic framework for Cr(VI) removal from aqueous solution, Desalin. Water Treat. 2020, 184, 139–149.
28. Wu, S. B.; Ge, Y. J.; Wang Y. Q.; Chen, X.; Li, F. F.; Xuan, H.; Li X. Adsorption of Cr(VI) on nano Uio-66-NH2 MOFs in water *Environ. Technol.* **2018**, *39*, 1937-1948
29. Niknam Shahrak, M.; Ghahramaninezhad, M.; Eydifarash, M. Zeolitic imidazolate framework-8 for efficient adsorption and removal of Cr(VI) ions from aqueous solution, *Environ. Sci. Pollut. Res*. **2017**, *24*, 9624–9634.
30. Li, X.; Gao, X.; Ai, L.; Jiang, J. Mechanistic insight into the interaction and adsorption of Cr(VI) with zeolitic imidazolate framework-67 microcrystals from aqueous solution, *Chem. Eng. J.* **2015**, *274*, 238–246.
31. Lin, D.; Liu, X.; Huang, R.; Qi, W.; Su, R.; He, Z. One-pot synthesis of mercapto functionalized Zr-MOFs for the enhanced removal of Hg^{2+} ions from water. *Chemical Communications (Cambridge, England)* **2019**, *55*, 6775-6778.
